# The inter-contact interval: a new measure to define frequent attenders in primary care

**DOI:** 10.1186/1471-2296-14-162

**Published:** 2013-10-23

**Authors:** Johannes Hauswaldt, Wolfgang Himmel, Eva Hummers-Pradier

**Affiliations:** 1 Department of General Practice/Family Medicine, University Medical Centre, Georg-August-University, Humboldtallee 38, 37073 Göttingen, Germany

**Keywords:** General practice, Office visits, Physician-patient relations, Appointment and schedules, Workload

## Abstract

**Background:**

Frequent attenders receive much attention in primary care research. Defining frequent attendance is crucial for an adequate view on this group of demanding patients. We aimed to develop a purely contact-based definition of “frequent attendance” and to apply it to real patients.

**Methods:**

From electronic records of 123 general practices in Germany, patients’ inter-contact intervals (ICI) between two consecutive doctor-patient-contacts were calculated in this retrospective observational study. ICI less than 7 days were labelled “frequent attendance”, patients with 60% or more of such intervals “frequent attenders (new view)”. In contrast, patients having at least 24 contacts per calendar year were considered “frequent attenders (traditional view)”. Both groups were analysed in their diseases and demands, using multiple logistic regression.

**Results:**

A total of 177,057 patients with at least 3 ICI in 1996 until 2006 yielded 4,408,033 ICI. One third were “short” ICI (less than 7 days), resulting in 19,759 (11.2%) frequent attenders (new). In contrast, 22,921 (12.9%) patients were frequent attenders (traditional). Compared to non-frequent attenders, frequent attenders (new) were more likely to have pneumonia (OR 1.66), stroke (OR 1.49), dementia (OR 1.46), or severe substance abuse (OR 1.44), also to need home visits or emergency attention. Frequent attenders (traditional) were more likely to have dementia (OR 2.76) or stroke (OR 2.06), and by far to need home visits (OR 5.43; all p < 0.001).

**Conclusions:**

A new measure, the interval in days of two consecutive face-to-face contacts (ICI), widens our perspective on frequent attenders in general practice. In many cases, their consultation behaviour and need for medical services seem to follow “disease logic”.

## Background

Frequent attenders in general practice receive great attention in primary care research because they demand high amounts of time resources, manpower, technical equipment and money. In spite of a large body of research, there are still many questions. It remains unclear which patients belong to the group of frequent attenders: the so-called “difficult patient”, making the general practitioner sigh “Oh no, not him again [1]!”, or patients with a psychosocial burden [2,3]? Are frequent attenders mainly those with chronic diseases or multimorbidity, or patients with a persistent problem, bedridden, in need for palliative care [4-7]?

Another key issue is how to handle the quantitative definition and measurement of frequent attendance. So far, frequent attendance is measured by counting contacts between patient and doctor within an arbitrary time period of 1 year, 2 years, or others. Different cut-off points for the definition of frequent attendance are recommended, for example the top 3%; or the top 10% of patients, or even more [6,8]. Some authors (e.g. [6]) recommend to stratify patients into several age groups per gender before analysing for frequent attenders. However, arbitrarily defined time periods for counting contacts seem to be inadequate to define frequent attendance appropriately, because this definition is based on the absolute number of contacts. It may be far more appropriate to consider the time period between two, or a series of, consecutive contacts as a running measure.

The aim of this study was to develop an inter-contact time measure, intending a new definition of “frequent attendance” and “frequent attenders”. In a second step, this measure was applied to real patients, focusing on their disease profile as well as the services they requested, and the results were compared with those achieved by traditional measures of frequent attendance.

## Methods

### Study design

In a retrospective study we analysed patient contact frequencies and time intervals between contacts. The data base was electronic patients records (EPR) from German general practices, between 1996 and 2006.

### Practices and patients

We used data from 123 general practices in three federal states of Germany (Lower Saxony, Baden Wurttemberg and Bremen) having participated in a large research project, which intended, among others, to use primary care EPRs for clinical or health service research (for details see [9-11]). Additional practices were recruited between 2005 and 2007. Participating practices gave informed consent to extract de-identified patient data that covered a defined time period.

### Data extraction and preparation for analysis

A study assistant with IT training visited all participating practices and extracted the requested patient data making use of a mandatory interface of the practice software program [11], as such data extraction via Internet is not compliant with current German legislation. EPR data were extracted once between 2002 and 2007, covering time periods between 2 and 11 years, de-identified for patient information immediately on practice premises and encrypted using a generic Java tool. These data were then stored in a SQL-database and prepared for further analysis [12]. Data refinement for this study excluded incomplete data cases (whole case analysis).

On daily base, face-to-face contacts between patient and physician were identified from fee-for-service data. Same data were also used to identify eight groups of medical services, such as full examination, electrocardiographic exam, home visits, lab tests etc. ICD-10 codes were truncated to three significant leading positions.

Eighty diseases, as indicated by about 3.500 ICD-10 codes, are officially declared as being “severe diseases” in the German statutory health insurance system and used for balancing the risks between insurances. The 20 most frequent of these 80 diseases were identified in our data set.

### Identification of frequent attenders

**Figure 1:**
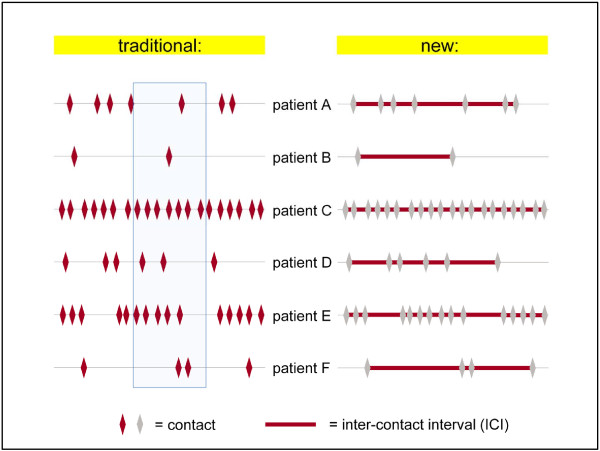
From contacts (traditional view) to inter-contact intervals (ICI, new) in exemplary patients.

Inter-contact intervals (ICI) were generated, calculating the time lag in days between every two consecutive face-to-face contacts of a patient with the doctor. To give an impression of how this measure yields different results than a mere counting of contacts in a given time period, consultation behaviour of exemplary patients is shown in Figure 1. Typically, traditional measures to define frequent attendance focus on the marked area on the left side of Figure 1, representing an additional reference time period chosen arbitrarily. While it seems rather clear that patient C and E are frequent attenders, patient A may also be a frequent attender when we consider his or her complete consultation behaviour over time instead of narrowing the focus on the fixed reference time period. As shown on the right side of Figure 1, the new approach shifts focus on each individual interval between any two consecutive contacts. Thus, ICI relates each contact to its own basic time measure, derived from the immediate time span since the previous contact, measured in days.

In order to define a patient as frequent attender, we first examined different cut-off values from our sample data for “short” ICI and different cut-offs for the minimum fraction of such ICI per patient. Based on these different outcomes we then decided for adequate cut-offs to define “frequent attendance” and “frequent attenders (new)”. To compare this new measure with existing traditional measures, we labelled a patient “frequent attender (traditional)” when he or she had 24 or more face-to-face contacts per calendar year. This measure had been derived at earlier stage of analysis from our crude sample data representing approximately 5% of patients with the highest number of annual contacts (95-percentile).

### Statistical analysis

We compared these two different measures of frequent attendance, i.e. being a frequent attender (new) or a frequent attender (traditional), on patient level by calculating Cramer’s V, a measure of contingency between two dichotomous variables, as displayed for example in a 2×2-table.

The association between the 20 most frequent severe diagnoses as well as 8 groups of medical services and being a frequent attender (new) or being a frequent attender (traditional) was modelled, calculating the adjusted odds ratios (OR) with their 99% confidence intervals (CI). Results are displayed as Forest plots.

## Results

The participating 123 practices provided data records from 362,163 patients with a total of 4,866,761 face-to-face contacts between patient and physician. Patients with one or two ICI in total were considered to be seeing the doctor sporadically only and excluded from further analysis. This left 177,057 patients with 4,408,033 ICI. The observed ICI values ranged from 1 to 3,556 days, with a median value of 11 days and an inter-quartile range from 4 to 32 days, and showed a strongly left-skewed density distribution (data not shown).

**Figure 2:**
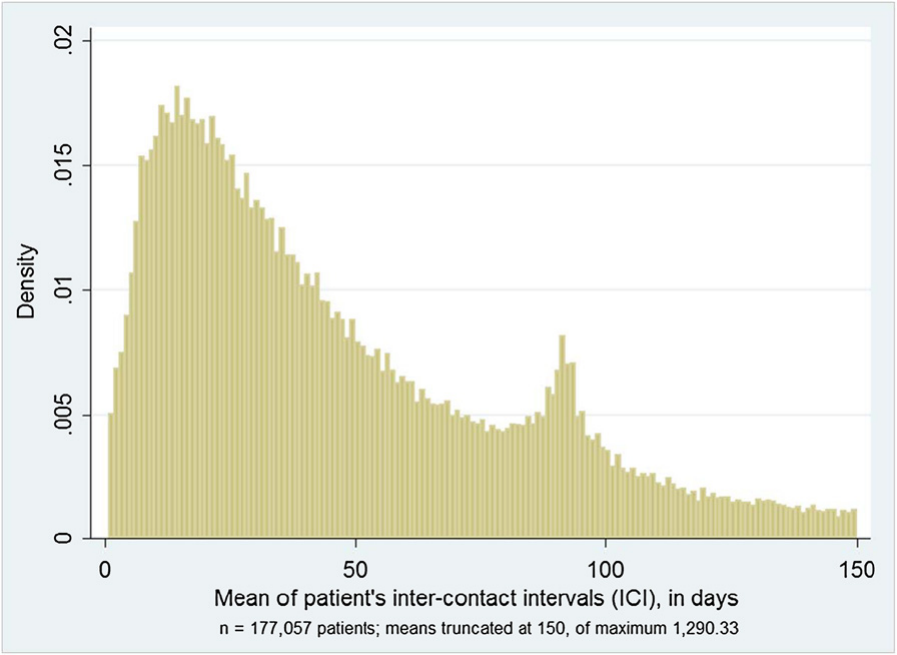
Density distribution of patient’s mean inter-contact interval (ICI), in days.

ICI median values on patient level had a mean of 37.3 (SD 58.6) days. Patients’ ICI mean values averaged to 64.1 (SD 78.5) days. The mode value (absolute maximum) of patients’ ICI means density distribution was found at the 14 days interval (Figure 2); a relative maximum in addition can be seen near an interval length of 90 days, likely to be due to quarter-annual visits, which are typical within the German prescribing and remuneration system. In addition, multiples of seven days peak up as relative maxima (Figure 2).

### Different cut-offs to define frequent attenders

**Figure 3:**
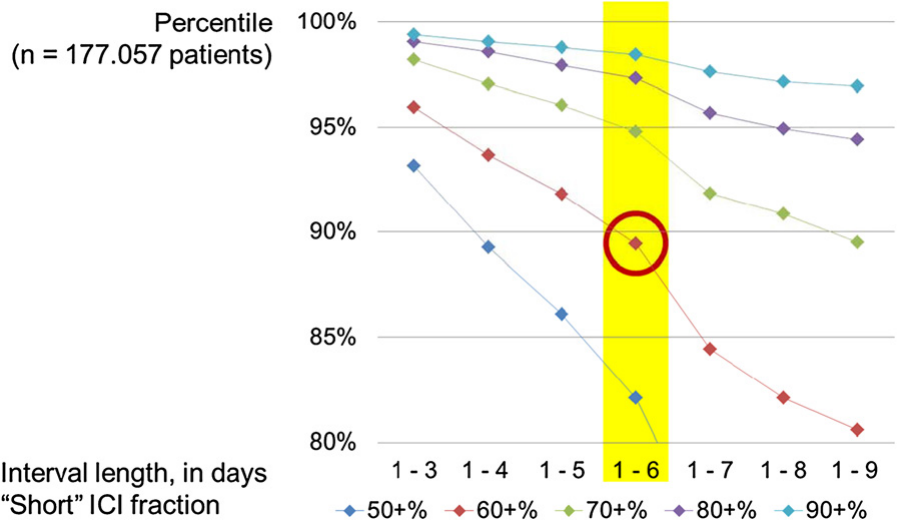
Observed patients’ percentile for several “short” ICI cut‒offs and their patient‒wise fraction.

To define a new and more adequate measure of frequent attenders we had to balance different cut-offs for “short” consultation intervals and for the fraction of such intervals on patient level. As can be seen in Figure 3, we examined cut-offs for “short” time periods between consultations from “1 to 3” up to “1 to 9” days, and cut-offs for percentages (fractions) of short periods on patient level from 50% up to 90%. Variation of these two cut-offs resulted in more or less patients being identified as “frequent attenders (new)”. Selecting an interval of “1 to 6” days to define a “short” ICI, together with the cut-off at 60% or more of these consultations found in a patient was considered a good compromise in specificity and practicability representing roughly those 10% of all patients which see their GP frequently, namely within a week (Figure 3).

### Frequent attendance and frequent attenders

About one third of all ICI (1,606,729 of 4,408,033) fell within a period of 1 to 6 days. A total of 19,760 patients (11.2%) had these short ICI in 60% or more of their total ICI and thus were labelled “frequent attenders (new)”. Using the more traditional definition of 24 or more annual contacts in at least one calendar year, 22,921 (12.9%) patients were “frequent attenders (traditional)”. Both characteristics, i.e. being a “frequent attender (new)” and a “frequent attender (traditional)”, were simultaneously present in 4,186 patients only, while 10.6% of the patients were “frequent attenders (traditional)” but not “frequent attenders (new)” and 8.8% were “frequent attenders (new)” but not “frequent attenders (traditional)”. The vast majority of 138,562 (78.3%) patients were not frequent attenders, neither according to the new nor to the traditional view. Congruency between the two measures, traditional and new, was therefore very low with a Cramer’s V of 0.0870 (p < 0.001).

### Diagnoses and medical services for frequent attenders

**Figure 4:**
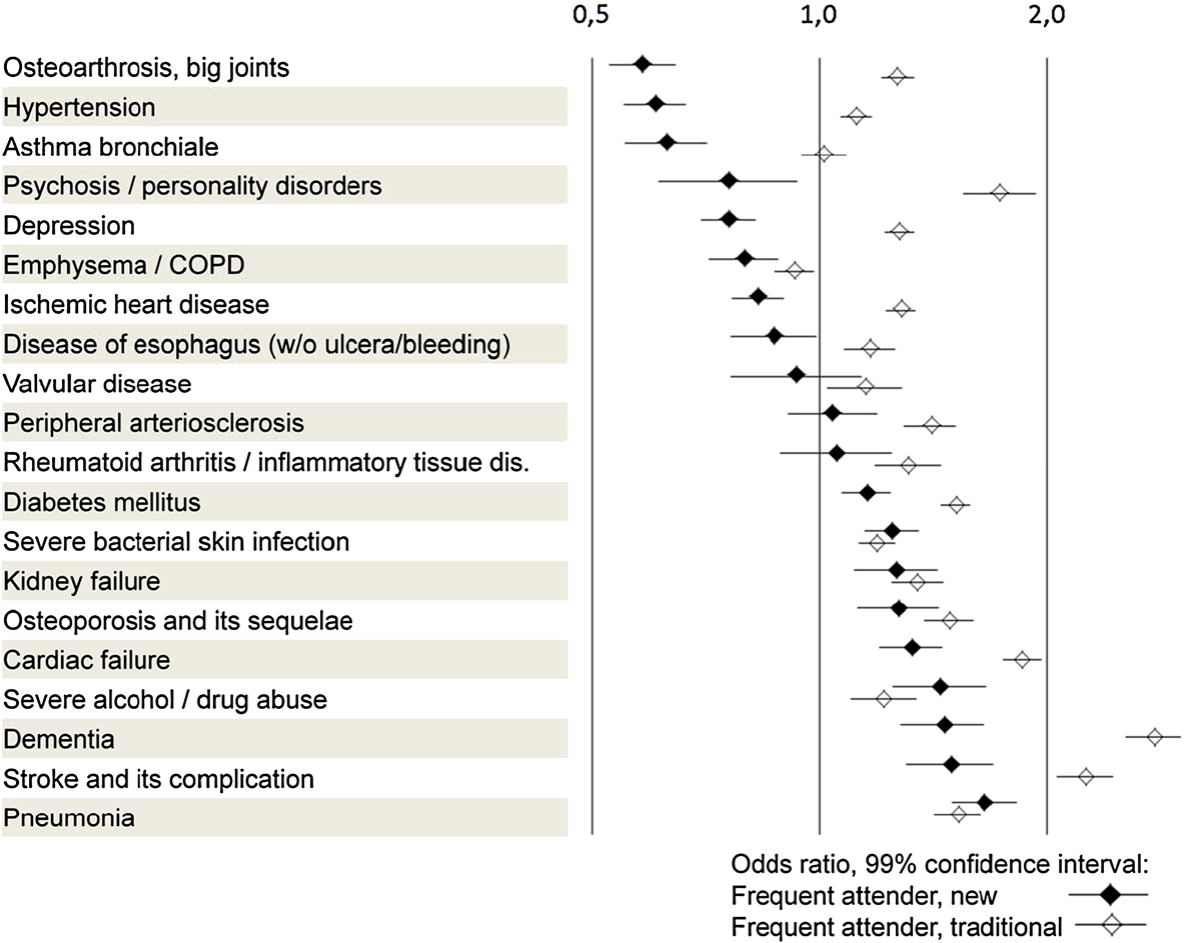
Association of frequent attender status with 20 severe diseases.

Figure 4 shows the diagnoses of “frequent attenders (new)” and “frequent attenders (traditional)” compared to their respective complements, non-frequent attenders. Frequent attenders (new) were more likely to have pneumonia (OR 1.66; 99% confidence interval 1.50 to 1.83), stroke (OR 1.49; 1.30 to 1.70) and dementia (OR 1.46; 1.28 to 1.65). Interestingly, they were less likely to be diagnosed with osteoarthritis (OR 0.58; 0.53 to 0.64), hypertension (OR 0.60; 0.55 to 0.71) or asthma (OR 0.63; 0.55 to 0.71).

In contrast, frequent attenders (traditional) were far more likely to have diagnostic labels in nearly all areas than frequent attenders (new), e.g. dementia (OR 2.76; 2.54 to 3.00), stroke (OR 2.76; 2.54 to 3.00), cardiac failure (OR 1.86; 1.75 to 1.97), and psychosis (OR 1.73; 1.55 to 1.93). Except for emphysema/COPD, diagnoses examined in this study occurred more often in frequent attenders (traditional) than in their complement (Figure 4).

**Figure 5:**
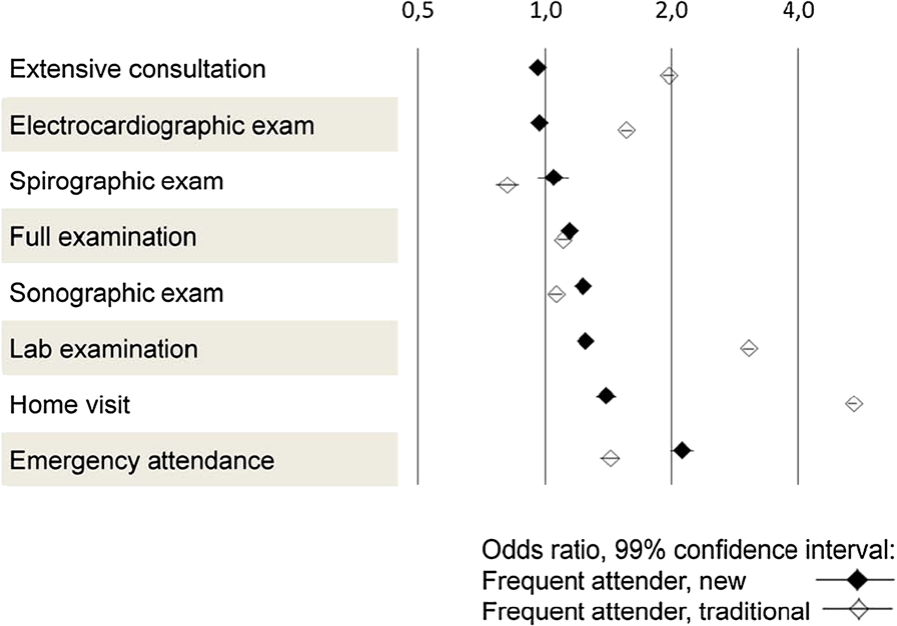
Association of frequent attender status with eight care groups.

As to medical services, frequent attenders (new) had more or less the same profile as non-frequent attenders (Figure 5)―they only needed more emergency attendances (OR 2.13; 1.99 to 2.26). In contrast, frequent attenders (traditional) received far more home visits (OR 5.43; 5.25 to 5.62) and laboratory tests (OR 3.04; 2.95 to 3.13).

## Discussion

### Summary of main findings

Traditionally, “frequent attendance” and “frequent attenders” are defined counting patient contacts within an arbitrarily defined time period. A new measure, the inter-contact interval, which is the time period between two consecutive contacts measured in days, is free from any arbitrary assumption and easy to apply for diagnoses’ and demand analysis. It may also be more appropriate for advanced analysis.

Applying the ICI as a new measure on routine data from EPR of 123 German general practices over 11 years, we identified 11.2% of all patients as “frequent attenders (new)”, compared to 12.8% of the patients being “frequent attenders (traditional)”. The overlap between these two groups was very low, suggesting that they represent clearly distinct patient populations.

### Strengths and limitations of the study

The participating practices were a “convenience sample” and thus may be subject to selection bias at practice level [10]. We included patients for whom the periods of observation ranged from 2 to 11 years. This may be surprising in a study that focused on contact frequency. However it is the very advantage of the new measure that we could apply it to a heterogeneous sample of patients, since the ICI relates each contact to its own basic time measure, derived from the immediate time span since the previous contact, measured in days. This has several advantages: (1) the basic time measure is not chosen arbitrarily but derived from the data itself. No limiting assumption is added. (2) As the unit of time measure is one day, the granulation is much finer than a basic unit of 1 year or so. (3) No implicit assumption is needed as to the minimum length of total patient observation time, which in the traditional approach has to be at least one period of the basic unit, i.e. 1 year, 2 years, etc. To obtain meaningful results, we chose a minimum of 3 ICI for a patient to be included.

The study is based on electronic patient records including reimbursement data, mandatorily comprising diagnoses, so that our analysis seems reliable. Availability of a large data sample allowed for a valid comparison between different definitions of frequent attendance. Moreover, since we had information about diagnoses as well as service demands of the patients included, we could show which diagnoses and medical services were associated with both definitions of frequent attenders.

External validity concerning the ICI is supported by detecting relative maxima at multiples of seven days and at about 90 days in patients’ averages of our sample (Figure 2), the latter being typical within the German prescribing and remuneration system, where statutory health insurance funds reimburse on a quarter-annual basis, and long-term prescriptions are issued mostly for 3 months [13,14]. In addition, slightly higher values than expected from straight lines at the ICI of 1 to 6 days (indicating less patients) and lower values at the ICI of 1 to 7 days (indicating more patients), respectively, as seen in Figure 3 may be explained by GPs who when checking-out patients may say “see you in one week again” rather than “see you after six days”.

When choosing 1 to 6 days to indicate a “short” ICI, the 60% cut-off allows approximately 10% of the patients in our sample to be labelled as “frequent attenders (new)” (90-percentile), while a 70% cut-off would yield about 5% (95-percentile) and an 80% cut-off about 2.5% (97.5-percentile) of all patients as “frequent attenders (new)”. We therefore consider these two generic cut-offs to well balance specificity and generalizability and thus to be most appropriate to describe frequent attendance in German primary care.

In spite of the nested character of our dataset (different patients in different practices), we decided not to perform a multilevel analysis: We neither intended to compare practices or patients, nor to analyse whether frequency of contacts can be explained by practice or patient characteristics. Moreover, since the number of practices included in our dataset is relatively large, it seems unlikely that our results are biased by distinctive practice characteristics or consultation behaviour, i.e. “random effects” of some practices or patients. We also wanted to avoid distracting from the basic idea of describing frequency of contacts and inter-contact intervals.

### Comparison with literature and clinical implications

Using the ICI as a new measure for identifying frequent attenders had the advantage of directly retaining more of relevant information which is in the original data than a mere counting of contacts and then relating the results to a general time period which has to be defined arbitrarily. Measuring days between two consecutive contacts was considered to be more appropriate as this lead to a quasi-natural definition of “frequent” attendance. It is the “rhythm” of attendance that matters in general practice and not an arbitrary definition of time period as applied in many studies e.g. [1-8,15-17].

Both identifiers―the new and the traditional definition of frequent attenders― showed overlapping in only few patients. Contingency measure between these two identifiers was very low and signalled only poor congruency between them [18,19].

Nevertheless, both identifiers simultaneously highlighted stroke, dementia, and cardiac failure as diseases being strongly associated with frequent attendance to primary care practice. Both measures pointed at association with demands that are especially time consuming and difficult to plan in general practice, namely emergency attendance with frequent attenders (new), and home visits with frequent attenders (traditional). This―and not so much breaches in communication or missing accord between patient and doctor―may be one reason why GPs sometimes complain about frequent attenders [1].

Even more interesting are the differences between the two groups of frequent attenders. Particularly, diagnoses as hypertension or diabetes mellitus characterize frequent attenders in traditional views, obviously because they require regular routine follow-ups, but are not a true indication of patient-created demand [20]. Our new measure for frequent attenders no longer highlights these diagnoses as typical for frequent attenders. On the contrary, hypertension is yet an example for low frequency rates, according to our new definition.

It is not surprising that people with psychiatric or psychological diagnoses are perceived as difficult patients [1-3]. Using the previous, more traditional definition of frequent attendance, one may suppose it is frequent consultations that may deter GPs. Our results on basis of the new definition, however, show that the “rhythm” of their consultations is not challenging more than that of other patients.

Emergency visits are another good example that our new measure for frequent attendance leads to meaningful results. An emergency visit is typically followed by several additional consultations―and exactly this is reflected in a high odds ratio for this service (Figure 5). In contrast, high odds ratios for home visit when using the traditional measure for frequent attenders are a characteristic of patient needs and not a psychological characteristic that may bother the GP.

### Future research

To better understand the implications of the ICI as a new measure, further research is needed when applying it to different patient settings of other countries. Re-analysis of existing data may also be helpful.

Koskela et al. [15]. propose to distinguish between temporary and persistent frequent attendance, as well as Smits et al. [16] and Morriss et al. [17] focus on persistent frequent attenders. Using the ICI in the way described in this paper served as an overall measure of a patient’s frequent attender status, but further analysis of a patient’s ICI for local ICI aggregation and dispersion may be helpful, for example using non-linear symbolic sequence analysing methods to detect ICI clusters ([21,22], see [23] for additional references), and for identifying episodes of care.

In addition, the cut-offs derived from our study data, though appropriate for German general practice, may have to be adapted to the primary care situation in other countries. For example, we chose a cut-off at “6 days” for “frequent attendance”, allowing a patient to show up every week without being identified as frequent attender. This cut-off mirrors the German background, where a high average number of visits to general practice is observed as compared to other countries. Our measure allows a flexible adaptation to other conditions in countries with other cultural peculiarities and different systems characteristics. The new measure facilitates comparison of analyses from different authors and gives a more precise picture of frequent attendance and its changes over time.

## Conclusion

No doubt, consultation behaviour varies, with some patients being frequent attenders, and indeed, there are associations between contact frequency and diseases. This is the rationale for the new measure ICI: to realize that some patients usually described as “frequent attenders” attend adequately, due to disease-related reasons. Also, it may be misleading to conclude a problematic or even a psychologically deviant behaviour (solely) from high consultation rates. The ICI measure leads to the general impression that visits to general practitioners follow a “disease logic” rather than a patient’s (or a physician’s) internal psychodynamic urge [1]. Thus, our new measure and the corresponding results may be a warning against premature conclusions: the physician, when realizing the internal feeling “oh no, not him again!”, should critically focus on his relationship to the patient or on flaws in their communication behaviour than on certain diagnoses or demands.

### Ethical approval

This study was approved by the local Ethical Committees of the University of Göttingen and the Hannover Medical School.

## Competing interests

The authors declare that they have no competing interests.

## Authors’ contributions

JH prepared and refined working data sets from primary data, also modelled care groups from re-imbursement data. JH, WH and EHP together developed the concept of inter-contact intervals from contact frequencies and designed concept verification by applying it on the working data. JH and WH drafted the manuscript; EHP participated in text emphasis and appropriate wording. All authors read and approved the final manuscript.

## Pre-publication history

The pre-publication history for this paper can be accessed here:

http://www.biomedcentral.com/1471-2296/14/162/prepub
